# Neurological, neurobehavioral, and radiological alterations in patients with mucopolysaccharidosis III (Sanfilippo's syndrome) in Brazil

**DOI:** 10.3389/fneur.2022.968297

**Published:** 2022-11-17

**Authors:** Daniel Almeida do Valle, Mara Lúcia Schmitz Ferreira Santos, Bruno Augusto Telles, Mara L. Cordeiro

**Affiliations:** ^1^Faculdades Pequeno Príncipe, Curitiba, PR, Brazil; ^2^Instituto de Pesquisa Pelé Pequeno Príncipe, Curitiba, PR, Brazil; ^3^Department of Child Neurology Hospital Pequeno Príncipe, Curitiba, PR, Brazil; ^4^Department of Radiology Hospital Pequeno Príncipe, Curitiba, PR, Brazil; ^5^Department of Psychiatry and Biological Behavioral Sciences, University of California, Los Angeles, Los Angeles, CA, United States

**Keywords:** mucopolysaccharidosis III, neurologic manifestations, epilepsy, magnetic resonance imaging, Sanfilippo syndrome

## Abstract

Mucopolysaccharidosis type III (MPS III) or Sanfilippo syndrome is the most common form of MPS, in which neurological involvement in all stages of the disease is prominent. The current study aimed to comprehensively describe the neurological profile of children and adolescents with MPS III who visited the largest pediatric hospital in South America. A prospective/retrospective cohort analysis was performed on 10 patients with MPS III from eight unrelated families. Most patients <12 months of age had achieved development milestones within the expected range for their age, with delay in walking independently and first single word acquisition. Behavioral symptoms were reported in seven patients. Eight patients (80%) developed profound intellectual disabilities. Six patients (60%) had epilepsy, among whom 75% had their first seizure between 2 and 4 years of age; the frequency of which increased with age. Monotherapy was effective in 60% of patients. Two patients, both aged <8 years, had normal baseline electroencephalographic activity. Epileptiform activity was observed in three patients. Cortical atrophy was visualized using magnetic resonance imaging in 71% patients; all but one of these patients were aged >6 years. Neurological abnormalities increased in prevalence and severity with age. Anti-seizure drug resistance was uncommon. Dysmorphological and systemic manifestations were uncommon and mild and did not correlate with neurological involvement. Despite high allelic heterogeneity, neurodegeneration was similar among all patients. Overall, these data contribute to the scarce literature from developing countries.

## Synopsis

Mucopolysaccharidosis type III is a group of lysosomal, neurodegenerative diseases that present with prominent neurological involvement in all stages of the disease. This study seeks to better understand the behavioral, neurophysiological, and radiological correlations in patients with these conditions. A better understanding of the neurologic symptoms, especially in behavior and epilepsy, may allow for better symptom management and improved morbidity.

The emergence of neurological and behavioral manifestations and loss of developmental milestones correlates with the appearance of radiological and electroencephalographic changes in patients with MPS III.

## Introduction

Mucopolysaccharidoses (MPS) are a group of rare inborn errors of metabolism caused by a deficiency of the lysosomal enzyme that affects the catabolism of glycosaminoglycans (GAG) (1). Lysosomal enzyme deficiency causes accumulation of intracellular substances, which alters normal cell function (1). MPS are chronic, progressive, and multisystemic. GAG accumulation can start *in utero* and can cause fetal hydrops, intrauterine death, or skeletal changes such as thoracolumbar kyphosis at birth (2). GAG accumulation is progressive and can consistently cause clinical symptoms throughout life (2).

MPS III, or Sanfilippo syndrome, is a group of diseases characterized by the deposition of lysosomal heparan sulfate (HS); it involves dysfunction of one of the genes that encode lysosomal enzymes involved in the degradation of HS (2, 3). It is a progressive disease that usually occurs in three phases: (1) developmental delay, especially of language marks; (2) behavioral changes and sleep disorders; and (3) loss of mobility, swallowing disorders, and epilepsy (4). The initial development of such patients is apparently normal until the first stage of the disease; in the first stage, the children show slight developmental delays accompanied by somatic manifestations such as recurrent ear, nose, and throat or gastrointestinal diseases. Subsequently, behavioral difficulties, hyperactivity, and sleep disorders with progressive worsening are observed; in the last phase, the child presents with loss of intellectual processes and motor functions. MPS III typically involves neurocognitive changes (5, 6). The predominant characteristics include severe mental deficiency, neurological degeneration, multiple dysostosis, and mild dysmorphological alterations (7–11).

Despite its prominent neurological involvement at all stages, some aspects of MPS III remain poorly understood. Although epilepsy has a high morbidity and is a frequent finding in MPS III (26–52%), features of MPS-related epilepsy over the disease course remain limited, including electrophysiological patterns, electroclinical profiles, and drug responses (12, 13). As proposed by Barone et al., an early recognition of epilepsy associated with a careful monitoring of the electroclinical features in MPS III may be necessary for appropriate care and detection of disease progression (12).

Therefore, the current study aimed to construct comprehensive understanding of the neurological, neurobehavioral, and radiological alterations in children and adolescents with MPS III who visited the largest hospital for children in Brazil.

## Materials and methods

This observational, descriptive cohort study was followed at the Pequeno Príncipe Children's Hospital and the Pelé Pequeno Principe Research Institute and included children and adolescents diagnosed with MPS III. All aspects of this study were approved by our Ethics Committee (protocol number: 31816320.2.0000.0097). Parents signed a consent form for the use of all data and images. Families of the patients received genetic counseling regarding the 25% recurrence risk in their next pregnancy.

Patients underwent corresponding studies from January 2005 to December 2021, the period when the systematized registry of the child neurology department of the Hospital began. We investigated, using quantitative and qualitative analysis of GAGs, patients with intellectual disability, developmental delay or developmental regression, coarse facies, thick hair and hirsutism, hepatosplenomegaly, joint stiffness, hearing loss, frequent upper respiratory and ear infections, and inguinal and/or umbilical hernias. Patients with increased urinary GAGs and/or those with isolated increase in HS based on GAG electrophoresis were considered. All patients with the aforementioned metabolic alterations or clinical signs strongly suggestive of MPS, even with normal urinary GAGs, performed an enzymatic assay. Simultaneous enzyme panel testing of N-sulfoglucosamine sulfohydrolase, N-acetylglucosaminidase (NAGLU), heparan-alpha-glucosaminide N-acetyltransferase (HGSNAT), and N-acetylglucosamine-6-sulfatase was carried out in leukocyte samples obtained from patients; enzyme deficiencies were identified based on comparisons with standard normal activity values. Deficient or low enzyme activity in any of these enzymes along with normal activity of the other three enzymes and clinical signs of the disorder is consistent with diagnosis of the corresponding subtype of MPS III.

Dermal cells were collected using oral swabs, and multigene panel analysis of the *SGSH, NAGLU, HGSNAT*, and *GNS* genes was performed. Buccal swab samples were collected, and DNA was extracted from the samples for genetic analyses with probes for the target regions. Next-generation sequencing was performed using Illumina technology: alignment and variant identification were performed based on bioinformatics protocols using the GRCh38 human genome as a reference. The potential pathogenic variants and regions with inadequate sequencing depth were confirmed using automated Sanger sequencing, which was conducted with a genetic analyzer. The variants were described according to the nomenclature recommended by the Human Genomic Variation Society. Novel variants were classified according to the guidelines of the American College of Medical Genetics and Genomics (14) on the basis of very low allele frequency, compound heterozygosity with a pathogenic variant, residue evolutionary conservation, and biochemical results. New variants were checked in the Human Gene Variant Database (https://www.hgmd.cf.ac.uk/) and ClinVar database (https://www.ncbi.nlm.nih.gov/clinvar/). Mutations were grouped according to type (missense or non-missense). Mutations resulting in a frameshift or splicing modifications were considered to be potentially pathogenic. The pathogenicity of novel missense mutations was predicted using *in-silico* analyses.

### Development milestone and behavior

Developmental history was gleaned from clinician reports during visits. A developmental screening scale, Denver Developmental Screening Test II (15), was used in face-to-face consultations, associated with child observation and behavior reporting by family members. The Portuguese version of Swanson, Nolan, and Pelham Rating Scale (SNAP-IV) (16) was used to screen for attention deficit hyperactivity disorder (ADHD), whereas the Portuguese version of M-CHAT (17) was used to screen for autism spectrum disorder (ASD). For the diagnosis, the American Psychiatric Association's Diagnostic and Statistical Manual of Mental Disorders Fifth Edition (DSM-5) criteria were used (18) by child neurologists and neurodevelopmental specialists. Intelligence is complex and no single test can meticulously measure all aspects of intelligence. However, intellectual or developmental disability (IDD) can be characterized by a significant impairment of cognitive and adaptive functioning (19–21). Once participants older than 6 years of age were unable to complete a formal intelligence test, such as the Wechsler Intelligence Scale, intellectual status was determined by an experienced pediatric neurologist based on developmental examination, daily living activities, and language skills.

### Epilepsy and electrophysiological study

Cases were classified as focal onset, generalized onset, or unknown onset seizures and epilepsy according to the operationalized 2017 I.L.A.E. classification systems (22, 23). The term “seizure-free” was used for participants who were completely free of seizures and had no auras but still took antiepileptic drugs as of December 2021 or the date of death.

Electrophysiological studies were carried out using electroencephalography (EEG); each recording was conducted for 40 min. Recordings were performed while patients were asleep (spontaneous or drug-induced sleep) as well as while they were awake. The EEG electrode placement pattern was set as per the 10–20 International System instructions. Filters of 0.5 and 40 Hz were used. The following graph elements were quantified: sleep spindles (spatial location, spectral frequency, duration, symmetry, and hemispheric synchrony), presence of epileptic seizures, or acute graph elements (type and location).

### Brain image

Magnetic resonance imaging (MRI) and MRI spectroscopy were performed with a 1.5 T-MR unit (Signa Explorer, GE Medical Systems, Milwaukee, WI). T1-weighted images [echo time (TE)/repetition time (TR) 11 ms/550 ms] and T2-weighted images (TE/TR 93 ms/4,000 ms) were obtained; fluid-attenuated inversion recovery (FLAIR; TE/TR/inversion time 110 ms/10,000 ms/2,250 ms) and diffusion-weighted imaging (DWI; TE/TR: 105 ms/5,200 ms) were performed. Spectroscopic imaging (2D univoxel Probe-Press) was performed with long (144 ms) and short TEs (35 ms).

### Statistical analysis

Statistical analysis was performed using the Statistical Package for Social Sciences (SPSS) for Windows, version 22.0 (IBM Corp, Armonk, NY, USA). Descriptive statistical analyses were performed by calculating summary measures, taking into account the nature of the variables. Inferential analysis was performed using statistical tests relevant to the study (Chi Square, Fisher's exact test, and Student's *t*-test), with a significance level of *p* < 0.05.

## Results

In total, 54 patients were diagnosed with MPS and 10 patients with MPS III since 2005. The patients belonged to eight distinct and unrelated families. Family consanguinity was observed in two cases. The demographic, clinical, and neurobehavioral features of patients with MPS III are summarized in Table 1.

**Table 1:** Clinical findings of MPS III patients.

	**P-1**	**P-2**	**P-3**	**P-4**	**P-5**	**P-6**	**P-7**	**P-8**	**P-9**	**P-10**
Type	A	A	B	B	B	B	B	B	C	C
Family number	1	2	3	4	5	5	6	7	8	8
Sex	F	F	F	M	M	F	M	M	M	M
Age of onset (years)	2	1	0	3	0	0	1	3		
Age of diagnosis (years)	12	7	15	4	4	1	2	12	9	17
Consanguinity	+	-	-	+	-	-	-	-	-	-
Epilepsy	+	+	+	+	-	-	+	-	-	+
Age of onset of epilepsy (Years)	2	5	14	3	-	-	2	-	-	0
Number of anticonvulsants in use	1	4	1	3	-	-	1	-	-	1
Aggressiveness	+	-	-	-	-	-	-	-	+	+
Hyperactivity	-	+	-	-	-	-	+	-	-	-
Autistic features	-	-	-	-	+	-	-	+	-	-
Cognitive impairment	+	+	+	+	+	-	-	+	+	+
Macrocephaly	+	-	-	-	+	+	-	-	+	+
Hepatomegaly	+	-	+	-	-	-	-	+	-	+
Esplenomegaly	-	-	-	-	-	+	-	-	-	-

F, Female; M, Male; F, Frontal; T, Temporal; MF, Multifocal.

**Figure 1:**
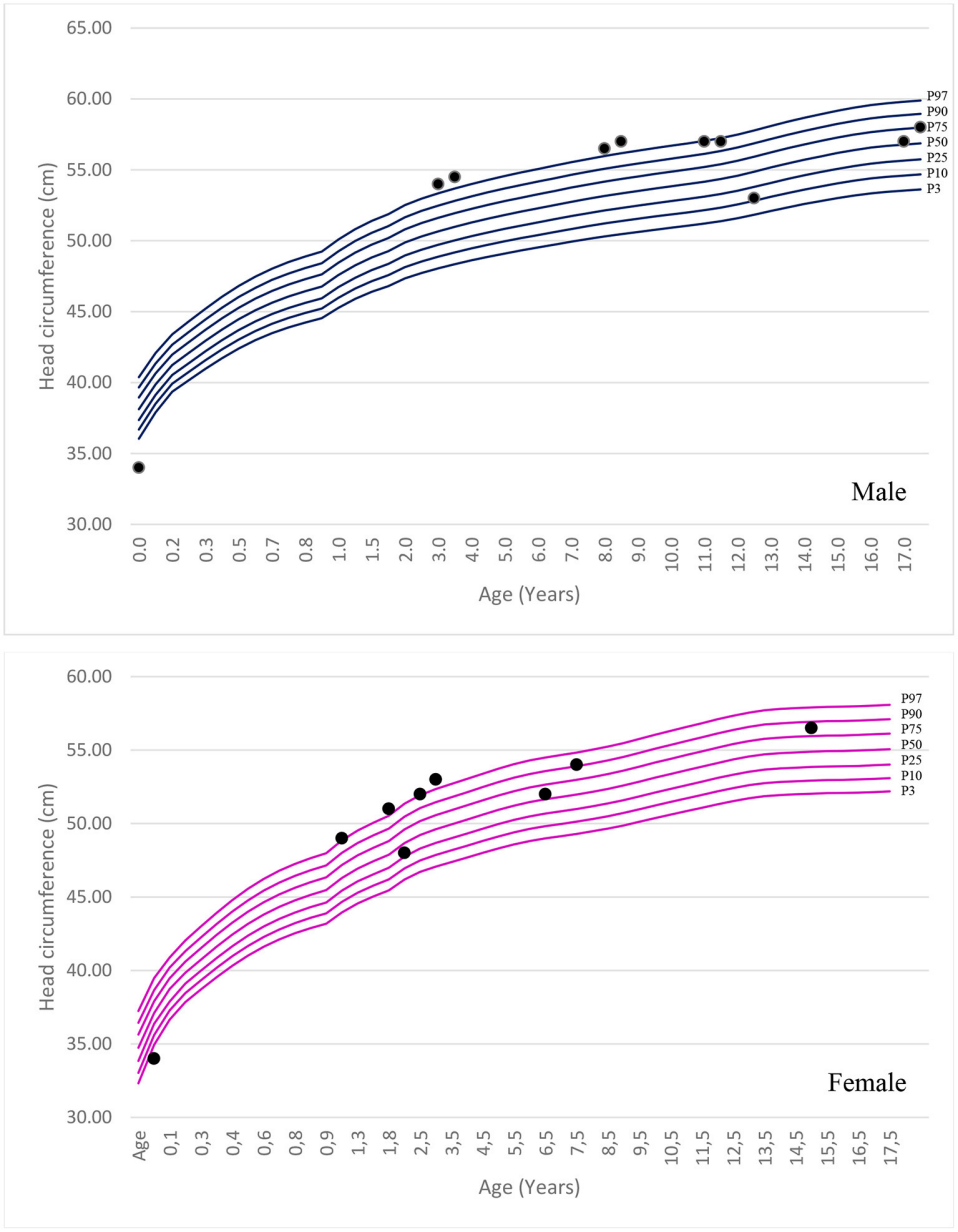
Head circumference according to age and sex in patients with MPS III.

The mean age of the patients in this study was 14 years (Interquartile Range (IIQ): 8–17 years). The median of first symptoms appearance was 1 year (IIQ: 0–2 years), and the median age at diagnosis was 8 years (IQR: 4–12 years). Two patients died at ages of 10 and 15 years. The urinary GAG level increased in 83% of cases (n=5/6), with a median of 235 (IQR: 195–406). Chitotriosidase was measured in two patients, and the results were within the normal range in both cases. Macrocephaly was identified in 50% of patients. The standard deviation change was observed in some patients after 1 year of age. Figure 1 shows the head circumference according to age.

Genetic testing was performed in five patients. Pathogenic variants were identified in four patients, whereas a variant of uncertain significance in homozygosity was identified in one patient. In one patient, the genotype was inferred through the study of their pedigree. Thus, genetic identification was possible in six patients, corresponding to five families (Table 2).

**Table 2:** Genetic variants identified by family.

**Gene**	**Category**	**Variant**	**Consequence**	**Position**	**Family**	**ACMG**
SGSH	Missense/Nonsense	c.220C>T	p.Arg74Cys	E2	2	P
	Microdeletion	c.1080delC	p.Val361fs	E8	2	P
NAGLU	Missense/Nonsense	c.419A>G	p.Tyr140Cys	E3	4	P
		c.503G>A	p.Trp168*	E3	6	P
		c.1004A>C	p.Tyr335Ser	E6	5	LP
		c.1693C>T	p.Arg565Trp	E7	5	P
	Indel	c.111_120delinsAGCC	p.Arg38_Leu40delinsAla	E1	7	VUS

LP, Likely pathogenic; P, Pathogenic; VUS, Variant of unknow significance.

### Development milestone and behavior

Most of the patients reached motor milestones within the expected time range for their age; however, a delay in the acquisition of unsupported gait was often identified (Table 3). Only two patients had no delay in language acquisition. Behavioral symptoms were reported in seven patients, one with aggressiveness and two each with associated ASD and ADHD. All but two patients developed profound intellectual disabilities during neurological follow-up, with severe impact on adaptive activities, dependence on others for all aspects of day-to-day life, and extremely limited communication ability.

**Table 3:** Median age of gain and loss of neuropsychomotor developmental milestones in patients with MPSIII.

**Development milestone**	**Median age with acquisition of milestones in months (IQR)**	**Median age for missed milestones in months (IQR)**
Cervical Support	3.5 (2.3–4.0)	-
Sit down unassisted	6.0 (6.0–8.3)	-
Walking independently	14.5 (13.8–16.5)	132.0 (72.0–132.0)
First single word	18.5 (12.0–24.0)	36.0 (25.5–42.0)

IQR, interquartile range.

### Epilepsy and electroencephalographic study

**Figure 2:**
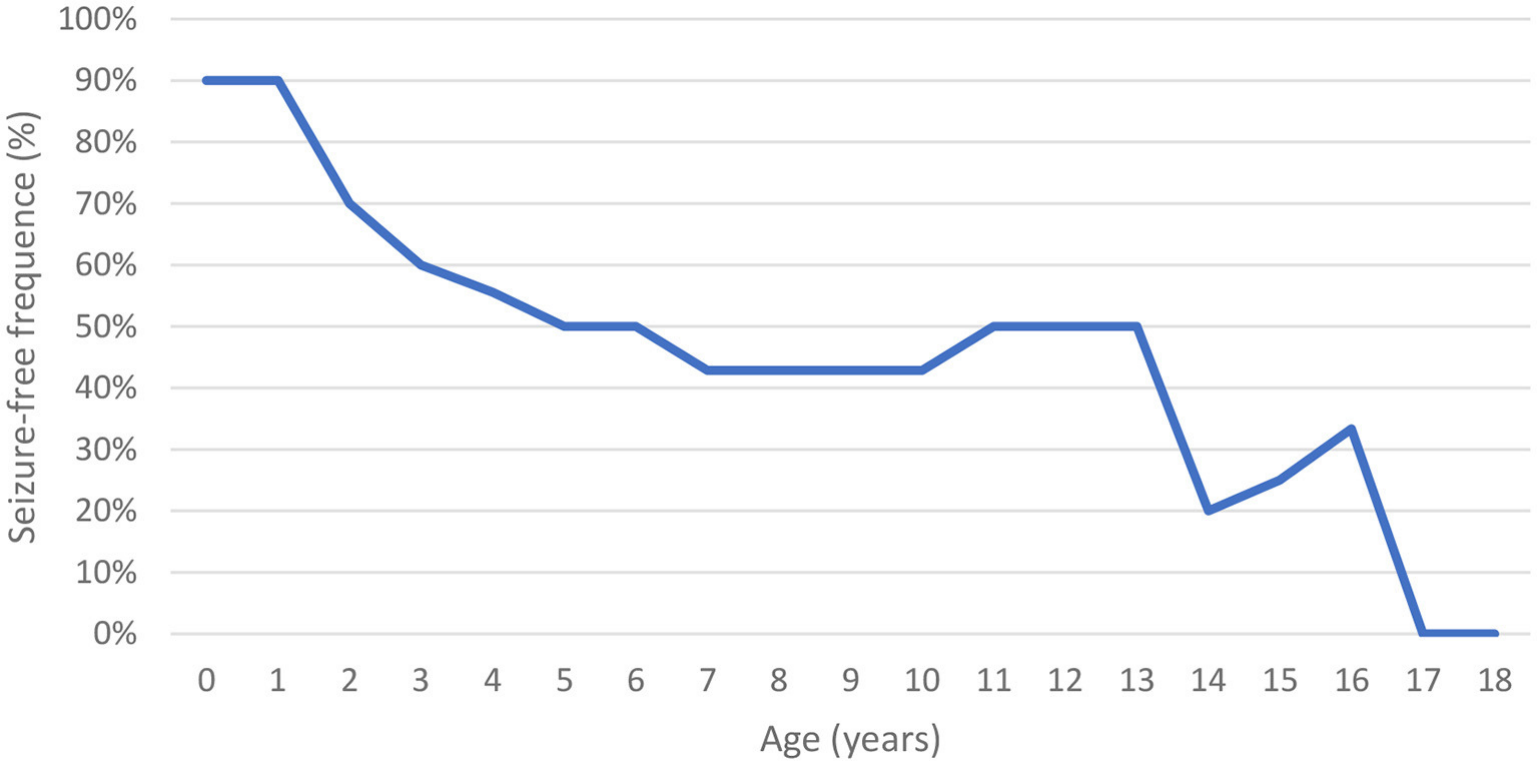
Seizure-free time in patients with MPS III frequency according to age.

Of the evaluated patients, 60% (*n* = 6/10) had epilepsy, and of these, 67% (*n* = 4/6) had their first seizure between 2 and 4 years of age. Of the seizure-free patients, three had MPS IIIB (aged 5, 8, and 17 years) and one had MPS IIIC (aged 15 years). Upon first seizure analysis, three patients presented with drop attacks (P-4, P-7, and P-10), two with focal emotional seizures with laughing (P-1 and P-2), and one with focal onset impaired awareness to bilateral tonic-clone seizure (P-3). The frequency of epilepsy increased with age, affecting approximately 40% of patients at 3 years of age (Figure 2). Two patients used more than one medication for seizure control, whereas 60% (*n* = 3/5) responded to monotherapy. Of the patients who used monotherapy, three were controlled with carbamazepine and one with sodium valproate. Of those on polytherapy, one presented control with the association of sodium valproate, carbamazepine, and clobazam, while the other with topiramate, clobazam, and oxcarbazepine.

An electroencephalographic study was performed in seven patients (Table 4). Three (P-4, P-7, and P-10) patients (43%) had normal baseline activity for their age, and all three were below the age of 8 years. Of these, two (P-4 and P-7) presented with disorganization in the follow-up EEG. The other patients had disorganized activity (P-1, P-2, P3, and P-8) with low voltage activity. Epileptiform activity was present in three patients (P-1, P-4, and P-7), all epileptic. Two of them presented with bursts of low-voltage fast activity and rhythmic spike pattern, predominant in the left temporal region, whereas one had an intense generalized epileptogenic activity, with greater frontotemporal intensity with presence of spike-ad-wave complex.

**Table 4:** Magnetic resonance imaging and electroencephalogram features of the patients with MPS III.

		**P-1**	**P-2**			**P-3**	**P-4**		**P-5**		**P-6**	**P-7**		**P-8**		**P-10**
MRI	Age at examination (years)	8	7	8		*	11	13	3	5	2	2	4	13		*
	Cortical atrophy	+	+	+			++	++	-	-	-	-		++	
	Ventricular enlargement	-	+	+		-	+	-	-	-	-	-	+	
	Enlarged perivascular spaces	-	-	-			+	+	-	-	-	-	+	+	
	White matter changes	-	-	+			+	++	-	-	-	+	+	+	
	Callosal atrophy	+	-	+			-	+	-	-	-	-	+	-	
	Basal ganglia involvement	-	-	-			-	-	-	-	-	-	-	-	
	Cerebellar changes	-	-	-			-	-	-	-	-	-	-	-	
	Thickening of the diploe	-	-	+			-	+	-	-	-	-	+	+	
	Spinal stenosis	-	+	+			-	+	-	*	*	*	-	-	
EEG	Age at examination	8	4	6	9	15	7	14	*		*	2	4	13	16	4
	EEG background disorganization	+	+	+	++	+++	-	+				-	++	++	++	-
	Type of epileptiform abnormality	Slow waves	-	-	-	-	Low voltage wave	Sharp wave				Low voltage wave	Spike-wave	-	-	-
	Localization	FT	-	-	-	-	T	F				T	G	-	-	-

-: absent; +: mild, ++: moderate; +++: severe; *not performed; EEG, electroencephalogram; F, frontal; G, generalized; MRI, Magnetic resonance imaging; T, Temporal.

### Brain image

**Figure 3:**
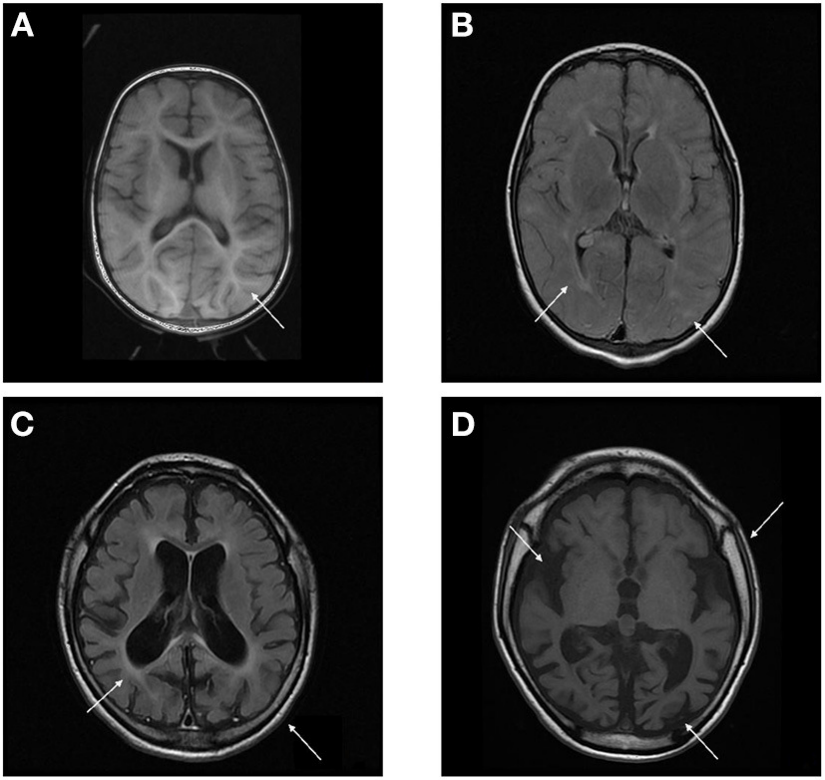
**(A,B)** Brain MRI T1 and T2/FLAIR sequence of P-7 showing absence of the usual myelination for the age group involving mainly the supratentorial compartment, especially in the anterior arm of the internal capsules. Multiple areas of high signal on T2 / FLAIR sparse across the white matter of the cerebral hemispheres and slightly asymmetrical, related to gliosis around perivascular spaces. **(C,D)** Brain MRI T1 and T2/FLAIR sequence of P-8 showing calvarial thickening and global accentuation of encephalic sulci and fissures, with relative infratentorial preservation and compensatory prominence of the ventricular system, denoting an important brain volumetric reduction. Multiple oval foci exhibiting high signal on T2 / FLAIR sparse through the white matter of the cerebral hemispheres and much of the confluent aspect.

The radiological abnormalities observed in this population are presented in Table 4. Cortical atrophy was visualized on MRI in 71% of patients (*n* = 5/7); all but one of these patients were over 6 years of age. Ex-vacuum hydrocephalus was observed in 29% (*n* = 2/7) of patients with clinical evolution and was mostly associated with cortical atrophy. White matter alteration and thickening of the diploe were identified in four patients each, and enlarged perivascular spaces were detected in three patients, with no relation to age or genotype. Spinal stenosis was observed in two patients. Figure 3 shows the brain MRI scans of participants P-7 and P-8, illustrating the main abnormalities identified.

## Discussion

In the present study, we evaluated the neurological profiles of ten patients with MPS III over a disease period ranging from 4 to 18 years. The median age at diagnosis was around 2 years (0–7), similar to that reported in the literature (24), and language delay was the most common first symptom. Due to the difficulty of access to services specialized in rare diseases, diagnosis is often late, and misdiagnosis, as in multiple other neurological conditions, occurs. As these symptoms are nonspecific and common to several neurological conditions, the diagnosis is often delayed when compared to other forms of MPS (25).

Few studies have assessed head circumference in MPS III. Macrocephaly was not a common finding when identified in the other forms of MPS. In this study, we observed macrocephaly in 50% of the patients; the frequency was similar to that reported by Ozkinay et al. (24). Furthermore, the literature describes that most patients present with hepatosplenomegaly caused by an accumulation of GAG (7, 24). In this study, hepatosplenomegaly was identified in 50% of the patients; the increase in liver size was often small.

MPS III has great molecular heterogeneity, and no common mutations have been identified. In this study, five different mutations in the *NAGLU* gene were identified in five patients from four families. Four of the mutations were missense mutations and one was an indel mutation, in line with that reported in the literature (24). Three families carried the same mutation on both alleles, and one family was compound heterozygous. Many patients had homozygous mutations, even in non-consanguineous families. A probable common ancestor can justify this finding, even though this assessment was not possible through pedigree analysis.

### Development milestone and behavior

The early neuropsychomotor development data in this study corroborated the findings of previous studies (26). The first motor milestones occurred in the normal time range, and an occasional slight delay in the acquisition of ability to walk independently was observed. In agreement with literature reports, which state that language development is usually delayed and occasionally occurs within the normal range (27), in this study, all but two patients showed delays in language development. Developmental delays and behavioral changes are the early symptoms identified by family members of patients with MPS III (25). Developmental delay was more evident in MPSIIIC when compared with the other forms. The delay becomes more evident in the period from 1 to 4 years of age, when there is a slowdown in the gain of development neuropsychomotor (DNPM) milestones (3, 28).

A sequential loss of milestones prevails, and the sequence of this loss is opposite to the sequence in which the milestones were gained (27). Developmental regression occurs after milestone acquisition stops and may be rapidly or slowly progressive in MPS III. Patients who experience an early presentation tend to have a greater speed of deterioration and cognitive decline, while those with a late onset have a slower evolution (29). Patients developing MPS tend to follow a progressive gain for up to 3–4 years, followed by loss of milestones related to the current age. The developmental regression in this age group does not allow patients to achieve the expected developmental gains in DENVER-II for ages 5 or 6 years. In this study, neuropsychological assessments were performed for some participants after the age of 6 years, but owing to the severity of cognitive impact, ground effects in the Wechsler scale test were observed. MPSIIIC presented a more indolent course, with a slower rate of loss of developmental milestones. The median age previously reported for loss of learned words was 7 years in all MPS III patients (27), which was later than that observed in this study, which was 3 years. This fact may correspond to an analysis bias, since the patients were followed up by developmental-behavioral pediatricians and child neurologists who may have identified the onset of loss earlier. In contrast, the median age of loss of unassisted sitting was previously reported as close to 11 years (26, 27).

In this study, cognitive impairment was present in most patients, and those without cognitive impairment were young. Except for those with severe cognitive impairment, all patients had behavioral symptoms. Behavioral symptoms have been reported to occur before 5 years of age in 74% and before 10 years of age in 94% of MPS III patients (30). The patients in this study exhibited ASD, ADHD, and aggressiveness. This is in line with previous studies that reported an association of MPS III with ADHD (frequency ranging from 46% to 95%) (24, 26, 31–34) and ASD (frequency ranging from 8 to 76%) (8, 35).

Central nervous system changes are cardinal in MPS III and manifest as intellectual disabilities, sleep disturbances, and hyperactivity (31, 36). In agreement with previous reports (29), severe and progressive neurodegenerations were observed in the current study. Neurological involvement occurs through different mechanisms and shows cumulative effects. Excessive GAG accumulation in the CNS leads to an increase in cerebellar dendritic cells and cytoplasmic neuronal distension. Likewise, the accumulation triggers neuronal degeneration and reduces neuronal density (37–40) as well as results in predominant gliosis in the white matter and thalamus (37, 38, 41), perivascular edema, and impairment of the integrity and functioning of the blood-brain barrier (42).

### Epilepsy and electroencephalographic study

The prevalence of epilepsy shows great variability among studies. Except for a Chinese study, which reported a low frequency of epilepsy (6%), the prevalence in other studies ranged between 36 and 79% (Table 5). In this study, the prevalence of epilepsy increased with age; most patients encountered their first seizures before the age of 10, concomitant with the deterioration of neurocognitive function (8, 12, 26, 27).

**Table 5:** Prevalence assessment, median age of onset and epilepsy pharmacoresistance in MPS III.

**Study**	**Country**	**Type of MPS**	**Epilepsy**		
			**Prevalence**	**Median age**	**Pharmaco-resistance**
(8)	France	All	40% (43/107)	*	*
		A	40% (30/73)	8.7 (3.2–13.7)	
		B	50 % (8/15)	8.8 (5.1–11.5)	
		C	31% (4/13)	(8–31)	
		D	17% (1/6)	9,4	
(16)	Spain	All	45% (25/55)	8.7 (2.5–37)	*
(17)	Sweden	All	79% (15/19)	*	*
(26)	Taiwan	All	36% (10/28)	11 (1.5–20)	*
(36)	Netherlands	A	66% (53/80)	11 (1–43)	21%
(12)	Brazil, Turkey, USA, Algeria, Azerbaijan, Canada, Colombia, Iran and Portugal	C	42% (8/19)	*	*
(37)	Netherlands	C	45% (10/22)	23 (16–33)	0%
(33)	Italy	All	53% (8/15)	10 (7–28)	13%
		A	71% (5/7)	10 (7–12)	0%
		B	40% (2/5)	9 (9–9)	20%
		C	50% (1/2)	28	50%
		D	0% (0/1)	0	0%
This study	Brazil	All	60% (6/10)	2.5 (2–4.5)	0%
		A	100% (2/2)	3 (2–5)	0%
		B	50% (3/6)	(2.5–8.5)	0%
		C	50% (1/2)	0	0%

*Data not available.

In our study, the main epilepsy type was generalized epilepsy, as previously reported (12, 13). Although epileptic seizures in MPS patients are reported as mainly tonic-clonic, the present study identified a difference in the pattern of first seizure (13). The most common seizure type in this study were drop attacks. Drop attacks are seizures that manifest as a sudden fall, lasting only one to a few seconds and causes injury. Generalized tonic, atonic, or myoclonic seizures can manifest with drop attacks. It is difficult to determine a specific seizure type for a “drop attack” based on history alone. Even with video recordings, a confirmation of a definitive seizure type cannot be made (43). These types of seizures, especially myoclonic seizures, are usually related to other lysosomal diseases, such as Galactosialidosis, Gaucher disease III, Gangliosidosis, Neuronal ceroid lipofuscinosis, Niemann-Pick C, and Oligosaccharidosis (2, 44). Although MPSIII is not usually associated with drop attacks, the high prevalence of generalized seizures in MPS III is interrelated to the strong association of myoclonic epilepsy with other lysosomal disorders, and the prevalence identified in this study points to the need for more investigations on myoclonic and atonic seizures in MPSIII. Nocturnal bouts of laughing or “panting,” currently classified by ILAE as focal emotional seizure with laughing, were relatively frequent. This has been described in the literature since 1993 (4) as uncommon and unusual. However, it currently has a higher frequency than previously expected, as identified in a recent study (12).

Electroencephalographic features with numerous abnormalities including encephalopathy and epileptogenicity (12, 31) as well as multifocal or diffuse white matter alterations (supported by postmortem histological evidence) (45, 46) are common in patients with MPS III. In this study, patients with EEG alterations had focal or multifocal discharges with variable disorganization of baseline activity, though ictal events were not recorded.

Despite being a neurodegenerative disease with structural changes, MPS III demonstrates uncommon anti-seizure drug resistance (Table 5) (30, 47), and most patients achieve good seizure control with monotherapy (26, 30, 48). No cases of seizure drug resistance were identified in the present study.

### Brain image

Cortical atrophy, which is a classic imaging finding in MPS III (24, 49–53), was identified in most patients in the current study. Cortical atrophy progresses with a compensatory increase in lateral ventricle size. In line with a study by Whitley et al. (53), cortical atrophy was observed over time, indicating a likely association between disease progression and radiological changes. Contrary to the findings reported by Zafeiriou et al. (52), white matter changes were not frequent, and progressive white matter changes were absent; myelination changes may have occurred at early ages and before the onset of developmental regression. Cystic changes (dilated perivascular spaces) in the corpus callosum, basal ganglia, and white matter were observed on MRI (54), but had low prevalence. This implies that though these changes are characteristic of the disease, they cannot be applied for radiological diagnosis.

The present study had some limitations. First, this was a single center study; the sample size was small and was, therefore, not representative of all patients with MPS III. Second, our findings were retrospectively and prospectively analyzed. Another limitation was the failure to carry out a genetic study in all patients participating in this study, due to operational problems. To our knowledge, this is the first study to assess neurologic and electrophysiological findings in MPS III in Brazilian/Latin American patients.

## Conclusion

Neurological, neurobehavioral, and radiological alterations in MPS III patients increased in prevalence and severity with age and were correlated with progressive neurological involvement. However, though MPS III is a neurodegenerative disease associated with structural changes, seizure drug resistance was uncommon. Dysmorphological and systemic manifestations were uncommon, mild, and did not correlate with neurological involvement.

The high allelic heterogeneity explains, in part, the increased phenotypic variability. Despite heterogeneity, the progression was constant in different patients. Many patients had homozygous mutations even in non-consanguineous families. A probable common ancestor can justify this finding, even though this assessment was not possible through pedigree analysis.

A careful neurological assessment, correlated with radiological and electrophysiological studies, may allow stratification of disease progression in MPS III.

## Data availability statement

The datasets presented in this study can be found in online repositories. The name of the repository and accession number(s) can be found below: National Center for Biotechnology Information (NCBI) ClinVar, https://www.ncbi.nlm.nih.gov/clinvar/, VCV000005108.36, VCV000518268.16, VCV000371634.8, VCV000092695.4, VCV000803393.1, VCV000001567.12, and VCV001700625.1.

## Ethics statement

The studies involving human participants were reviewed and approved by Human Research Ethics Committee of the Hospital Pequeno Principe (n. CAAE: 31816320.2.0000.0097). Written informed consent to participate in this study was provided by the participants' legal guardian/next of kin. Written informed consent was obtained from the individual(s), and minor(s)' legal guardian/next of kin, for the publication of any potentially identifiable images or data included in this article.

## Author contributions

DV conceptualized the research, gathered and analyzed the data, and wrote the initial manuscript. BT reviewed the neuroimaging scans. MC coordinated the study, revised, and critical reviewed the manuscript for key intellectual content. All authors contributed to drafting the manuscript, approved its final version, and agreed to be accountable for all aspects of the work.

## Funding

This study was funded in part by the Coordenação de Aperfeiçoamento de Pessoal de Nivel Superior (CAPES), Brazil (Finance Code 001) to DV.

## Conflict of interest

The authors declare that the research was conducted in the absence of any commercial or financial relationships that could be construed as a potential conflict of interest.

## Publisher's note

All claims expressed in this article are solely those of the authors and do not necessarily represent those of their affiliated organizations, or those of the publisher, the editors and the reviewers. Any product that may be evaluated in this article, or claim that may be made by its manufacturer, is not guaranteed or endorsed by the publisher.
